# Neutral Atom Imaging of the Solar Wind‐Magnetosphere‐Exosphere Interaction Near the Subsolar Magnetopause

**DOI:** 10.1029/2020GL089362

**Published:** 2020-10-05

**Authors:** S. A. Fuselier, M. A. Dayeh, A. Galli, H. O. Funsten, N. A. Schwadron, S. M. Petrinec, K. J. Trattner, D. J. McComas, J. L. Burch, S. Toledo‐Redondo, J. R. Szalay, R. J. Strangeway

**Affiliations:** ^1^ Southwest Research Institute San Antonio TX USA; ^2^ Department of Physics and Astronomy University of Texas at San Antonio San Antonio TX USA; ^3^ Physics Institute University of Bern Bern Switzerland; ^4^ Los Alamos National Laboratory Los Alamos NM USA; ^5^ Space Science Center University of New Hampshire Durham NH USA; ^6^ Lockheed Martin Advanced Technology Center Palo Alto CA USA; ^7^ Laboratory for Atmospheric and Space Physics University of Colorado Boulder Boulder CO USA; ^8^ Department of Astrophysical Sciences Princeton University Princeton NJ USA; ^9^ Institut de Recherche en Astrophysique et Planétologie Université de Toulouse Toulouse France; ^10^ Department of Electromagnetism and Electronics University of Murcia Murcia Spain; ^11^ Earth and Space Sciences University of California Los Angeles CA USA

**Keywords:** energetic neutral atoms, solar wind‐magnetosphere‐exosphere interaction, charge‐exchange

## Abstract

Energetic neutral atoms (ENAs) created by charge‐exchange of ions with the Earth's hydrogen exosphere near the subsolar magnetopause yield information on the distribution of plasma in the outer magnetosphere and magnetosheath. ENA observations from the Interstellar Boundary Explorer (IBEX) are used to image magnetosheath plasma and, for the first time, low‐energy magnetospheric plasma near the magnetopause. These images show that magnetosheath plasma is distributed fairly evenly near the subsolar magnetopause; however, low‐energy magnetospheric plasma is not distributed evenly in the outer magnetosphere. Simultaneous images and in situ observations from the Magnetospheric Multiscale (MMS) spacecraft from November 2015 (during the solar cycle declining phase) are used to derive the exospheric density. The ~11–17 cm^−3^ density at 10 R_E_ is similar to that obtained previously for solar minimum. Thus, these combined results indicate that the exospheric density 10 R_E_ from the Earth may have a weak dependence on solar cycle.

## Key Points

- ENA cameras image both magnetosheath and magnetospheric plasmas in the vicinity of the subsolar magnetopause
- Magnetospheric plasma is not distributed evenly across the dayside near the magnetopause
- The exospheric hydrogen density near the magnetopause may have a weak dependence on solar F10.7

## 1 Introduction—The Subsolar Magnetopause

The Earth's magnetopause separates magnetospheric and shocked solar wind plasmas. It is approximately a paraboloid of revolution around the Earth‐Sun line with a subsolar standoff distance of ~10 Earth Radii (R_E_) for 1.5 nPa solar wind dynamic pressure. Solar wind H^+^ that is slowed and heated across the Earth's bow shock is diverted around the magnetopause.

The closest approach to the Earth for magnetosheath plasma is the subsolar magnetopause. However, the magnetopause is not impenetrable to this plasma. Magnetic reconnection is the dominant process that allows transfer of plasma across the boundary. This plasma forms a boundary layer at the magnetopause and eventually travels through the Earth's cusps and into the magnetotail.

In addition to magnetosheath plasma entering the magnetosphere, other plasma populations are locally resident in the Earth's magnetosphere. One of these is a ~10s to 100s of eV population of H^+^ and higher energy O^+^ called the warm plasma cloak (Chappell et al., 2008). This population originates in the high‐latitude ionosphere and is often found near the noon/duskside magnetopause (Fuselier et al., 2017). Another population is a <1 to ~10s of eV population of H^+^ and He^+^ called the plasmaspheric plume. This population originates from the midlatitude to high‐latitude ionosphere and populates the plasmasphere surrounding the Earth. When magnetospheric convection is enhanced, a plume of plasmaspheric plasma convects to the dayside/duskside magnetopause. The warm plasma cloak and plume are distinguished by their energy and composition (Fuselier et al., 2017).

Fractions of these plasma populations charge‐exchange with the Earth's hydrogen exosphere, or geocorona, that extends beyond the Earth's magnetopause. The exospheric density, n_H_, decreases as ~(1/R^3^); therefore, ENA production in the charge‐exchange equation H^+^ + H^0^ → H_ENA_ + H^+*^ depends strongly on distance from the Earth. Here, H^+^ is a magnetospheric or magnetosheath proton, H^0^ is an exospheric hydrogen atom, H_ENA_ is a (neutral) ENA, and H^+*^ is a newly created, very cold (<1 eV) proton.

Before it charge‐exchanged, the parent ion for the H_ENA_ was gyrating around the local magnetic field. The newly created H_ENA_ is not bound by the magnetic field. Therefore, it propagates with the parent ion energy in the direction of its original gyromotion. These ENAs have been used to remotely image the shocked solar wind at the subsolar magnetopause (Fuselier et al., 2010) and in the magnetospheric cusps (Petrinec et al., 2011).

Fuselier et al. (2010) combined simultaneous remote ENA observations and in situ observations of magnetosheath ions near the subsolar magnetopause to determine n_H_ at the subsolar point, ~10 R_E_ from the Earth. The neutral hydrogen density ~10 R_E_ from the Earth was ~8 cm^−3^ for these observations obtained near solar minimum under relatively low F10.7 levels.

This paper reports the first simultaneous observations of ENAs and parent magnetospheric ions from near the magnetopause. It uses simultaneous remote ENA observations and in situ observations of shocked solar wind and magnetospheric protons to demonstrate that magnetospheric and magnetosheath ion populations are distributed differently along the line‐of‐sight of the ENA imagers. The magnetosheath population is used in combination with the ENA imaging and a model for the density and velocity of the magnetosheath plasma to determine n_H_ near the magnetopause for an interval in the declining phase of the solar cycle. The exospheric densities derived here and in Fuselier et al. (2010) for solar minimum conditions are similar, indicating that n_H_(10 R_E_) does not depend strongly on solar cycle.

## 2 IBEX and MMS Observations

ENA observations are from IBEX (McComas et al., 2009). IBEX was launched into Earth orbit in October 2008 to investigate the global interaction between the solar wind and the interstellar medium. It has two single‐pixel ENA cameras, IBEX‐Lo and IBEX‐Hi, that cover energies from 0.01 to 2 keV and 0.54 to 6 keV, respectively (Funsten et al., 2009; Fuselier et al., 2009). The cameras view perpendicular to the IBEX spin axis, and this axis is repointed toward the Sun twice per orbit. The ~9‐day orbit has an apogee of ~50 R_E_, which keeps the spacecraft well outside the Earth's bow shock for a large fraction of the time.

Twice a year in November‐December and March‐April, the ENA cameras' fields‐of‐view (FOV) include the subsolar magnetopause as they sweep through the ecliptic. Each 15‐s spin, the data are binned into a ring of 60 6° × 6° pixels. Each pixel contains the LOS integrated hydrogen ENA flux at eight energies for IBEX‐Lo and five energies for IBEX‐Hi. These single‐spin strips are combined and transmitted to ground as 92‐spin packets (spanning 23 min).

In combination with IBEX observations, in situ magnetosheath and magnetospheric plasma observations are from MMS. MMS is a multispacecraft mission launched into Earth orbit in March 2015 to investigate magnetic reconnection in the near‐Earth environment (Burch et al., 2016). The Hot Plasma Composition Analyzer (HPCA) (Young et al., 2014) is one of many MMS instruments. HPCA is a time‐of‐flight mass spectrometer that measures the full 3‐D plasma distributions for major solar wind and magnetospheric ion species (H^+^, He^2+^, He^+^, and O^+^) in 10 s. Science operations for the first phase of the primary mission began in September 2015, and through March 2016, the spacecraft apogee of 12 R_E_ swept through the dayside magnetopause from the dusk to dawn terminator. In November‐December 2015, the spacecraft apogee was near the subsolar point, providing many opportunities for conjunctions with IBEX remotely imaging the subsolar magnetopause while MMS observed the plasma in situ.

## 3 Observations on 4 November 2015

Figure S1 in the supporting information shows the IBEX and MMS spacecraft orbits projected into the X‐Y_GSE_ plane on 4 November 2015. At 0306 UT, MMS crossed the magnetopause very near the subsolar point. The magnetopause was compressed, with a subsolar standoff distance of 9.3 R_E_. For 6 hr surrounding the magnetopause crossing, IBEX was on the duskside at a distance of 45 R_E_ from the Earth. The 6.5° FOV of the ENA cameras included the subsolar magnetopause and regions in the magnetosphere, magnetosheath, and solar wind.

Figure S2 shows the solar wind conditions from 0100 to 0700 UT convected to the magnetopause. Up to 0400 UT, the solar wind dynamic pressure was steady at ~2 nPa. This pressure was somewhat higher than 1.5 nPa, which is why the magnetosphere was compressed. At 0400 UT, the magnetic field magnitude increased sharply by ~50%, and the solar wind density and dynamic pressure increased by more than a factor of 3. The higher pressure and density persisted for almost 2 hr.

**Figure 1:**
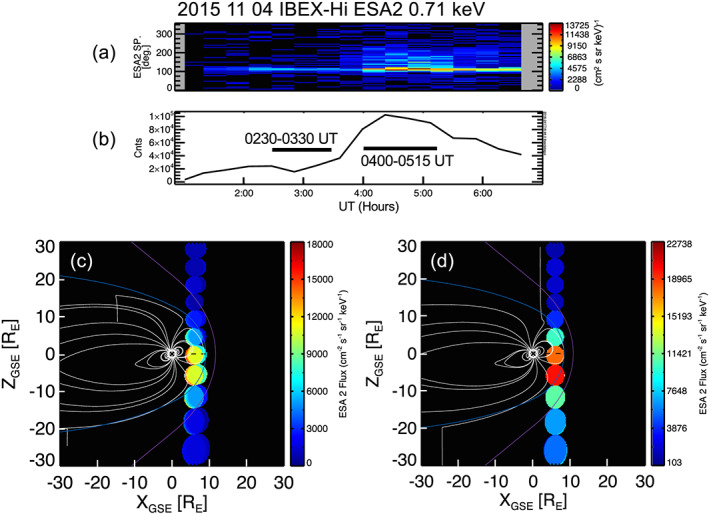
(a) Angle‐time spectrogram of IBEX ENA fluxes at 0.71 keV. (b) Corresponding counts summed over all angles from a showing the sharp increase after the solar wind compression at 0400 UT. (c and d) ENA images showing the projection of pixels in the noon meridian for the two intervals before (c) and after (d) the compression. Field lines in the noon‐midnight plane are from Tsyganenko (1995). Each ~6.5° × ~ 6.5° circular pixel contains ~Y_GSE_ LOS integrated fluxes (multiple pixels are overlayed with slight X offset). Fluxes increase dramatically after the magnetospheric compression.

Figure 1 shows some IBEX observations during this 6‐hr interval. Figure 1a shows a spin angle‐time spectrogram of the 0.71 keV flux (cm^2^ s sr keV)^−1^ from IBEX‐Hi. Fluxes at about 120° are when IBEX‐Hi viewed more‐or‐less the subsolar magnetopause. There is a clear flux increase from that direction at 0400 UT, coinciding with the dynamic pressure increase in Figure S2.

Figure 1b shows counts summed over all spin angles versus time. Counts increase by a factor of 3–4 from 0300 to 0430 UT. The absolute uncertainty in the IBEX‐Hi ENA flux is 20% (e.g., Fuselier et al., 2014) and counting statistics in IBEX‐Hi are excellent. Two intervals from 0230 to 0330 UT and from 0400 to 0515 UT are selected to represent presolar and postsolar wind compression, respectively.

Figures 1c and 1d show fluxes (cm^2^ s sr keV)^−1^ in the strip of 6.5° × 6.5° pixels centered on the subsolar magnetopause for the two intervals before and after the compression. The pixel with the peak flux is closer to the southern cusp and somewhat offset from the subsolar point. This effect is well known from previous images of the cusps and subsolar region for large dipole tilt (Petrinec et al., 2011). Across all IBEX‐Hi energies, the flux from close to the southern cusp is <20% higher than that in the pixel near the subsolar point for both precompression and postcompression time intervals.

**Figure 2:**
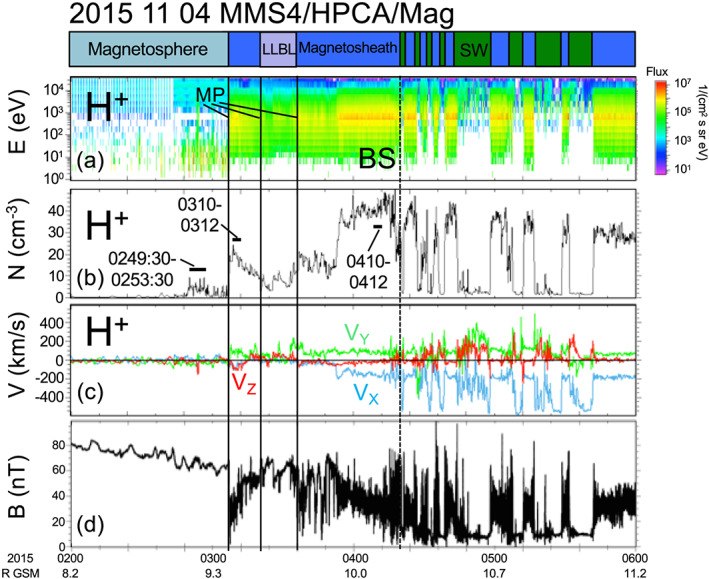
In situ plasma and magnetic field observations from the subsolar magnetopause region. (a) H^+^ energy‐time omni‐directional fluxes, (b) H^+^ density, (c) three components of the H^+^ velocity, and (d) total magnetic field. The first magnetopause crossing is at 0306 UT. While in the magnetosheath at 0350 UT, the solar wind compression arrives and the density increases by a factor of ~3. After the compression, the spacecraft crosses the bow shock and enters the solar wind. Three intervals in panel (b) are used to compare with IBEX ENA images.

**Figure 3:**
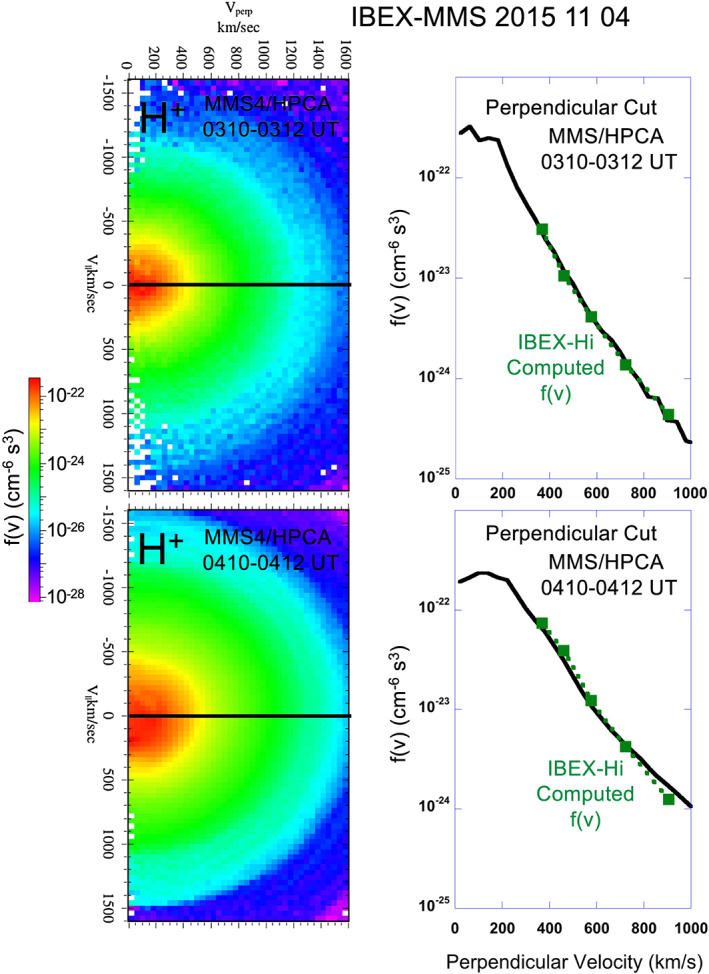
(Left‐hand panels) 2‐D H^+^ pitch angle distributions for pre‐ and post‐compression periods. The distributions are in the rest frame of the H^+^ distribution. (Right‐hand panels) 1‐D cuts in the H^+^ distributions perpendicular to the magnetic field. These ion distributions are compared to the ion energy distribution computed from the IBEX‐Hi ENA distributions. The two energy distributions are nearly identical, indicating that the average ion energy distribution along the IBEX LOS is well‐represented by the ion energy distribution near the subsolar point measured by MMS.

Figure 2 shows 4 hr of in situ plasma and magnetic field observations from the MMS4 spacecraft. The spacecraft is very close together, and MMS4 was chosen for these in situ measurements. Figure 2a shows a plasma region identifier. The spacecraft is in the magnetosphere from 0200 to 0306 UT. There is a high energy (~1 to 40 keV) magnetospheric ring current population as well as a lower energy (few eV to 200 eV) population identified as a combination of warm plasma cloak and plasmaspheric plume. The spacecraft crosses the magnetopause 9.3 R_E_ from the Earth at 0306 UT and observes magnetosheath H^+^ fluxes from ~0.005 to 40 keV that peak at ~0.8 keV. The spacecraft crosses the magnetopause twice at 0322 and 0336 UT and then returns to the magnetosheath, with similar magnetosheath fluxes as at 0306 UT. In the magnetosheath up to 0354 UT, V_X_ is small because the plasma has no radial velocity near the subsolar point. The solar wind compression arrives at MMS4 at 0354 UT and the H^+^ density increases by nearly a factor of 3. V_X_ decreases to −200 km/s, indicating that magnetopause is rapidly receding earthward. The first bow shock crossing at 0420 UT confirms this earthward motion. After 0420 UT, the spacecraft crosses the bow shock multiple times before returning to the magnetosheath at 0540 UT; 0310–0312 UT and 0410–0412 UT are chosen to represent the in situ magnetosheath plasma at the subsolar point presolar and postsolar wind compression, respectively. H^+^ distributions averaged over these two intervals are shown in Figure 3. Similar fluxes to those in the interval from 0310 to 0312 UT were observed when MMS reentered the magnetosheath at 0336–0338 UT. A third interval, 0249:30–0253:30 UT, is chosen to represent the in situ magnetospheric plasma before the solar wind compression.

## 4 Combined ENA and In Situ Observations: Computing the Exospheric Density

The in situ proton and hydrogen ENA fluxes are related by (1).

(1) $$J_{\mathrm{ENA}}\left(E,x,z\right)=\int J_{\mathrm{ion}}\left(E,x,y,z\right)\sigma \left(E\right)n_{H}\left(x,y,z\right)\mathrm{dl}$$

Here, *J*_*ENA*_(*E, x, z*) is the column integrated ENA flux that depends on energy and the *x,z* Geocentric Solar Ecliptic (GSE) coordinates, *J*_*ion*_(*E, x, y, z*) is the magnetosheath/boundary layer ion flux that depends on GSE coordinates, σ(*E*) is the energy‐dependent charge‐exchange cross section (Lindsay & Stebbings, 2005), *n*_*H*_(*x, y, z*) is the exospheric hydrogen density, and the column integral is computed along the l direction, which is a function of Y_GSE,_ Z_GSE_.

For the precompression and postcompression time intervals, (1) is converted to a sum of 6.5° × 6.5° by 1 R_E_ cylindrical elements along the IBEX line‐of‐sight (LOS) for the pixel closest to the subsolar point. There are 21 cylindrical elements from approximately Y = +10 R_E_ to Y = −10 R_E_. The middle cylindrical element at the subsolar point contains the MMS in situ measurement of *J*_*ion*_(*E, x, y, z*). Geometries of the precompression and postcompression intervals are similar, and geometries of the 21 elements for the precompression interval are in the supporting information.

Each of five IBEX‐Hi energies provides an independent measure of *n*_*H*_ because the exospheric density depends only on distance from Earth. In each Δl = 1 R_E_ cylindrical element, the Chamberlain model (1/R^3^ falloff) is assumed for *n*_*H*_ (e.g., Chamberlain, 1963; Collier et al., 2005; Rairden et al., 1986):

(2) $$n_{H}=n_{H0}R_{0}^{3}/{\left(x^{2}+y^{2}+z^{2}\right)}^{3/2}$$

Here, *n*_*H*0_ is the exospheric density at *R*_0_ = 10 R_E_.

The ion flux *J*_*ion*_(*E, x, y, z*) in each cylindrical element is anchored at the subsolar point by the MMS measurements; however, a model is needed for this quantity at other locations. Figure 3 shows that the functional form (i.e., temperature) of the ENA and ion energy distributions are nearly identical. This result implies that *J*_*ion*_(*E, x, y ,z*) at the subsolar point effectively represents the energy distribution at any point along the IBEX LOS. Therefore, *J*_*ion*_(*E, x, y, z*) along the LOS depends on the local density and velocity toward or away from IBEX. Although there are other possible models for the magnetosheath gasdynamic parameters (see the supporting information), the normalized densities and velocities along the integral pathlength were determined from the Spreiter et al. (1966) model, with the magnetopause location adjusted to the precompression and postcompression standoff distances. In addition, a 0.5 R_E_ thick boundary layer inside the magnetopause with properties of the magnetosheath was assumed. This boundary layer was observed from 0322 to 0336 UT in Figure 2.

Using these normalized values, *J*_*ion*_(*E, x = subsolar standoff distance,y = 0, z = 0*) observed by MMS was scaled by the normalized density, and the flux was adjusted higher or lower depending on the magnetosheath flow toward or away from IBEX, respectively. The final flux reduction accounts for the percentage of the cylindrical element in the magnetosphere or solar wind (see the supporting information).

Sample parameters for the integration and the resulting values for *n*_*H*0_ for the precompression and postcompression periods are in the supporting information. Averaged over five independent measurements from five IBEX‐Hi energy channels, *n*_*H*0_(10 R_E_) for the precompression and postcompression intervals were 11 ± 2 cm^−3^ and 17.5 ± 3.5 cm^−3^, respectively. The uncertainty is primarily due to the 20% uncertainty in the IBEX‐Hi absolute flux. The standard deviation of the mean is less than 20%, indicating that *n*_*H*0_(10 R_E_) is independent of IBEX‐Hi energy. The somewhat higher *n*_*H*0_(10 R_E_) postcompression may be because the in situ magnetosheath measurement was not at the subsolar point and therefore may be an underestimate of ion fluxes at the magnetopause. Alternatively, the somewhat higher *n*_*H*0_(10 R_E_) postcompression may be due to fast exospheric density response to the compression, as seen in Lyman‐alpha observations (Zoennchen et al., 2017)

**Figure 4:**
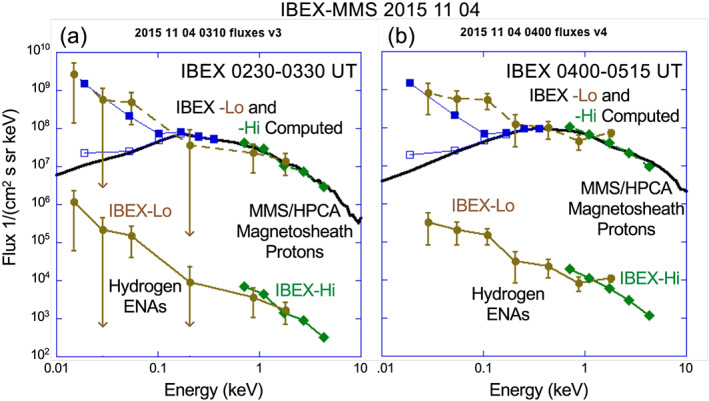
MMS H^+^ and IBEX hydrogen ENA fluxes before (a) and after (b) the solar wind compression. Lower curves show IBEX ENA fluxes. Black curves show MMS magnetosheath proton fluxes and blue squares show MMS magnetospheric proton fluxes. Open blue squares are average values and solid blue squares are peak values. Two upper curves (brown for IBEX**‐**Lo and green for IBEX**‐**Hi) are proton fluxes computed from ENA fluxes. Computed and observed proton fluxes agree very well. After the compression, the agreement between IBEX‐Lo computed fluxes below 0.1 keV is not as good because there were no in situ magnetospheric observations from MMS after the compression.

Figures 4a and 4b show IBEX ENA fluxes, MMS/HPCA proton fluxes from 0.01 to 10 keV, and computed proton fluxes using the ENA fluxes for all IBEX‐Lo and IBEX‐Hi energy channels. IBEX‐Lo channels that had no counts above background are not shown. The energy‐dependent propagation time for ENAs from the subsolar magnetopause to IBEX is accounted for. The average values for *n*_*H*0_ from above were used to match the ENA and ion fluxes. The ~10^3^ difference between the ENA fluxes and the ion fluxes at energies above 0.1 keV indicates that only ~0.1% of the magnetosheath protons undergo charge‐exchange in the magnetosheath (see also Fuselier et al., 2010; Ogasawara et al., 2013).

Figures 4a and 4b show that magnetosheath proton fluxes observed by MMS and computed proton fluxes from the IBEX observations agree very well for energies from 0.1 to 6 keV. Below 0.1 keV, computed proton fluxes are much higher than magnetosheath proton fluxes. Open blue squares in Figure 4 show the addition of average magnetospheric proton fluxes over the 4‐min time interval from 0249:30 to 0253:30 UT, and the solid blue squares show peak magnetospheric proton fluxes. Observed peak fluxes match computed fluxes much better than observed average fluxes. HPCA underestimates magnetospheric proton fluxes for very cold populations (Toledo‐Redondo et al., 2019); however, for intervals in Figure 4, the population is not extremely cold, and this instrumental effect does not account completely peak and average flux differences.

## 5 Discussion and Conclusions

The link between the ENA fluxes below 0.1 keV and the magnetospheric plasma near the magnetopause demonstrates imaging of low‐energy magnetospheric plasma for the first time. That is, the main contribution to ENA fluxes >0.1 keV is magnetosheath H^+^ while the main contribution to ENA fluxes <0.1 keV is low‐energy magnetospheric H^+^. Previous ENA and H^+^ observations from the subsolar region extended from about 6 keV down to 0.1 keV and therefore did not image the magnetospheric population (e.g., Ogasawara et al., 2013). Furthermore, observations in Figure 4 show that, unlike the energy distribution of the magnetosheath plasma, the functional form of the average energy distribution of the magnetospheric plasma below 0.1 keV is not well‐represented by the in situ energy distribution at the subsolar point or, equivalently, the magnetospheric plasma is not distributed quasi‐uniformly along the integral LOS of the imager. In contrast, Figure 3 shows that the functional form of the ENA and in situ magnetosheath ion distributions are nearly the same above 0.1 keV. In addition, plasma fluxes below 0.1 keV derived from ENA imaging must be considerably higher than average fluxes measured in situ at a single point and time by MMS. Nonuniform distribution of the low‐energy magnetospheric plasma has been observed as variations in density time series measured in situ (e.g., Fuselier et al., 2017). Higher fluxes may be related to the fact that the pixels span several R_E_ and low‐energy magnetospheric plasma is sampled closer to the Earth than 8–9 R_E_. After the solar wind compression, the computed flux from the ENAs at 0.1 keV is much higher than even the peak flux observed in situ. This difference is consistent with a compression and/or heating of magnetospheric plasma near the magnetopause. In situ observations <0.1 keV were only available prior to the compression because, after the compression, MMS4 was in the magnetosheath and solar wind. Differences before and after the compression demonstrate the ability to image large‐scale magnetospheric density changes in response to changing solar wind conditions.

Under the assumptions detailed in the previous section, ENA fluxes are consistent with n_H_(10 R_E_) = 11 ± 2 cm^−3^ precompression and n_H_(10 R_E_) = 17.5 ± 3.5 cm^−3^ postcompression.

Fuselier et al. (2010) used observations from IBEX‐Hi and in situ magnetosheath observations from the Cluster spacecraft to determine n_H_. They report densities ~8 cm^−3^ (four events with densities from 4 to 11 cm^−3^) at 10 R_E_ from the Earth using a different method for determining n_H_.

Observations in Figures 1, 2, 3, 4 were made in November 2015, during the declining phase of the solar cycle with solar F10.7–110 sfu. Observations in Fuselier et al. (2010) were made in 2009, during solar minimum. In March‐April 2009, F10.7 was ~70 sfu. F10.7 levels for 2015 observations were about 50% higher than those in 2009, yet there is at best a small increase in the exospheric density. Thus, the combination of results from 2015 with those from Fuselier et al. (2010) suggest that n_H_(10 R_E_) may have a weak dependence on F10.7.

Exospheric densities from 8–10 R_E_ have been estimated using two other techniques. Rairden et al. (1986) and later Zoennchen et al. (2015) and Baliukin et al. (2019) used scattered geocoronal Lyman‐alpha under solar minimum and solar maximum conditions to model n_H_. The Zoennchen et al. (2015) model was not valid beyond 8 R_E_; therefore, densities at larger distances are extrapolated and have large, essentially unknown uncertainties. Because uncertainties *R* ≥ 8 R_E_ are not quantified, it is difficult to determine solar cycle variation of n_H_. Considering Figure 10 of Zoennchen et al. (2015), n_H_ at 9 R_E_ may be ~15 cm^−3^ for solar minimum and ~40 cm^−3^ for solar maximum, with unknown error bars. Zoennchen et al. (2015) found a decrease that was slower than *r* ^−3^. However, using their *r* ^−2.75^ in (2) results in <5% change in n_H_(10 R_E_). Finally, Baliukin et al. (2019) modeled densities on the flank magnetosphere at 10 R_E_ were about 20–50 cm^−3^. Uncertainties for these strongly model‐dependent densities are unknown.

A second, newer technique used observed X‐ray emissions from charge‐exchanged, high charge state solar wind oxygen in the magnetosheath and a global MHD model of the magnetosphere to predict n_H_ at 10 R_E_ (Connor & Carter, 2019). They predicted n_H_ ~40 and ~60 cm^−3^ for two events near solar maximum. The F10.7 levels for their events were 144 and 206 sfu, respectively. These are higher than the F10.7 levels for the event in Figure 4. However, it is difficult to compare results from the two techniques because their technique used X‐ray observations that were not optimally suited for imaging the subsolar magnetopause, and they did not have simultaneous, co‐located in situ observations of the high charge state ions. This technique used MHD simulation results validated with in situ proton density observations and high charge state oxygen content from a solar wind monitor (Whittaker & Sembay, 2016). The modeled magnetosheath and the low time resolution oxygen observations introduce uncertainties that are difficult to quantify. Thus, while X‐ray imaging is interesting and promising tool, a direct comparison with the observations in this paper must wait for a dedicated X‐ray instrument to image the subsolar magnetopause, simultaneous observations of proton distributions, and high charge state oxygen concentrations.

## Supporting information



Supporting Information S1

## Data Availability

IBEX‐Hi and IBEX‐Lo magnetospheric data are available online (http://ibex.swri.edu/researchers/publicdata.shtml#dr12). The MMS science data center (https://lasp.colorado.edu/mms/sdc/public/links/) has the MMS data. Solar wind data were obtained through CDAWeb (https://cdaweb.gsfc.nasa.gov/index.html/). Southwest Research Institute research was NASA funded through MMS contract NNG04EB99C, IBEX subcontract 80NSSC19K1107, and HGI grant NNX17AB98G. ISSI supported this study via the international *Cold plasma of ionospheric origin at the Earth's magnetosphere* team.
