# Supplementary material for: Neutral Atom Imaging of the Solar Wind‐Magnetosphere‐Exosphere Interaction Near the Subsolar Magnetopause
Source: Geophys Res Lett. 2020 Oct 5;47(19):e2020GL089362. doi: 10.1029/2020GL089362 (PMC7757190; doi:10.1029/2020GL089362)
Supplement: Supplementary file 1 — Supporting Information S1 [file GRL-47-e2020GL089362-s001.docx]

**Neutral atom imaging of the solar wind-magnetosphere-exosphere interaction near the subsolar magnetopause**

S. A. Fuselier^1,2^, M. A. Dayeh^1,2^, A. Galli^3^, H. O. Funsten^4^, N. A. Schwadron^5^, S. M. Petrinec^6^, K. J. Trattner^7^, D. J. McComas^8^, J. L. Burch^1^, S. Toledo-Redondo^9,10^, J. R. Szalay^8^, and R. J. Strangeway^11^

^1^Southwest Research Institute, San Antonio, TX, USA, ^2^Department of Physics and Astronomy, University of Texas at San Antonio, San Antonio, TX, USA, ^3^Physics Institute, University of Bern, Bern, 3012, Switzerland, ^4^Los Alamos National Laboratory, P.O. Box 1663 Los Alamos, NM 87545, USA, ^5^University of New Hampshire, Durham, NH, USA, ^6^Lockheed Martin Advanced Technology Center, Palo Alto, CA, USA, ^7^Laboratory for Atmospheric and Space Physics, University of Colorado Boulder, Boulder, CO, USA, ^8^Department of Astrophysical Sciences, Princeton University, Princeton, NJ 08544, USA, ^9^Institut de Recherche en Astrophysique et Planétologie, Université de Toulouse, CNRS, UPS, CNES, Toulouse, France, ^10^Department of Electromagnetism and Electronics, University of Murcia, Murcia, Spain, ^11^Earth and Space Sciences, University of California, Los Angeles, CA, USA

**Supplemental Material:**

Contents of File: Text, two tables and three figures.

**S1. Spacecraft Locations and Solar Wind Conditions on 4 November 2015**

Figure S1 shows the locations of the IBEX and MMS spacecraft on 4 November 2015. IBEX was near its orbit apogee and the subsolar magnetopause was in the field-of-view (FOV) of the imagers. The subsolar magnetopause was 44 R_E_ from IBEX in nearly a direct line along the Y_GSE_ direction. MMS was on its outbound trajectory and crossed the magnetopause at 0306 UT at (9.3, 0.7, -0.5 R_E_) GSM.

Figure S2 shows the solar wind conditions surrounding the solar wind compression. The omni data shown has been convected to the Earth’s bow shock. The solar wind was relatively steady before the compression. The ~2 nPa dynamic pressure is somewhat higher than the nominal pressure of ~1.5 nPa and the magnetopause subsolar location was observed by MMS to be 9.3 R_E_ from the Earth. The compression at 0400 UT moved the magnetopause earthward to a subsolar distance of 8.3 R_E_.

**S2. Integration of the ion flux along the IBEX LOS**

To determine n_H_ at 10 RE from the Earth, the integral in (1) in the paper is converted to a sum (Equation S1) along of 6.5ºx6.5º by Δl = 1 R_E_ cylindrical elements along the IBEX LOS for the pixel closest to the subsolar point in Figure 1 of the paper.

$J_{ENA}\left( E, x, z \right)= \sum J_{ion}\left( E, x, y, z \right)\sigma\left( E \right)n_{H}\left( x,y,z \right)\Delta l$ (Equation S1)

Figure S3 shows the geometry of the 21 cylindrical elements from approximately Y = +10 R_E_ to Y = -10 R_E_ that are included in the sum for the pre-compression interval. The geometry is nearly the same for the post-compression interval, with the important difference that the magnetopause is at 8.3 R_E_ instead of 9.3 R_E_. The middle cylindrical element contains the in situ measurement of *J_ion_*(*E,x=9.3 R_E_,y = 0, z = 0*) from MMS at the subsolar point and is used to anchor the sum. Each element is described by 5 points as shown in Figure S3. These 5 points are used to determine the percentage of the element that is in the magnetosheath or 0.5 R_E_ thick boundary layer adjacent to the magnetopause.

For each cylindrical element and IBEX-Hi energy step, the following quantities are needed to determine its contribution to the sum in Equation S1: the average *J_ion_*(*E,x,y,z*) over the cylindrical element (the variation in this quantity over the element is <10%), the charge exchange cross section, σ(E), the percentage of the element that is in the magnetosheath or boundary layer, the distance from Earth of the part of the element that is in the magnetosheath or boundary layer (used in the Chamberlain model (2) in the paper).

The average ion flux *J_ion_*(*E,x,y,z*) in each cylindrical element is anchored at the subsolar point by the MMS measurements; however, a model is needed for this quantity. As pointed out, Figure 3 in the paper implies that the average ion energy distribution measured at the subsolar point effectively represents the energy distribution at any point along the IBEX LOS. Therefore, *J_ion_*(*E,x,y,z*) at any point along the LOS depends on the density and velocity toward or away from IBEX.

There are some possible models for the density and velocity in the magnetosheath along the IBEX LOS. One possibility is to use a global MHD simulation. However, this model has one very important aspect that is not consistent with the observations. The magnetosheath distribution function in an MHD simulation is a maxwellian, while the observed distributions in Figure 3 in the paper are distinctly non-maxwellian. In fact, the Maxwellian assumption underestimates the flux in the lowest two IBEX-Hi energy channels by up to a factor of 3. This flux underestimate for the lower energy channels would produce the unphysical result that *n_H_* depends on the IBEX ENA energy.

The normalized densities and velocities for each cylindrical element along the integral pathlength were determined from a magnetosheath gasdynamic model (Spreiter et al., 1966) with Mach number 8 and γ = 5/3. The Mach numbers estimated from the OMNI data set were 9.6 and 7.8 for the pre- and post-compression intervals, respectively. The magnetopause for the gasdynamic model was adjusted so that the standoff distance was 9.3 R_E_ for the pre-compression interval and 8.3 R_E_ for the post-compression interval. The bow shock location was also scaled from the Spreiter et al. (1966) model so that the subsolar standoff distances of the bow shock pre- and post-compression were 11.1 R_E_ and 9.9 R_E_, respectively. The bow shock location for the post-compression interval agrees reasonably well with the in situ MMS crossing at 10.2 RE at 0422 UT in Figure 2. In addition, a 0.5 R_E_ thick boundary layer inside the magnetopause with properties of the adjacent magnetosheath was assumed to exist. This boundary layer was observed by MMS at the subsolar point. Table S1 has the normalized densities and velocities and the other quantities for the 21 cylindrical elements for the 0.71 keV energy step. It is clear from Figure S3 and from the model ratio of the densities in Table 3 that the 21 cylindrical elements are located on the dayside magnetosphere relatively near the subsolar point. The change in density is relatively small from the center cylindrical element to the end cylindrical elements. In Table S1, there is a percentage of the cylindrical element that is in the magnetosheath/boundary layer. To compute this percentage, the GSE location of the center and edges of the cylinder were determined and compared to the model magnetopause and boundary layer location. This percentage ranged from 0% at Y~+10 R_E_ to ~90% at the subsolar point.

The sum of the right-hand column of Table S1 is the right-hand side of Equation S1, or J_ENAcomputed_(E, x, z)*n_H0_ = 5.23x10^2^ 1/(cm^2^ s sr keV)(cm^-3^). From Table S1, the ENA flux at 0.71 keV observed by IBEX was 5.65x10^3^ 1/(cm^2^ s sr keV). Dividing the first quantity by this ENA flux yields n_H0_ = 10.8 cm^-3^. It is interesting that nearly half of J_ENAcomputed_ comes from the first two cylindrical elements on the duskside magnetopause. The reason for this high contribution is that, although the first two cylindrical elements are relatively far from the subsolar point, the density is only 30% lower while the flux is substantially higher because the ion distribution is propagating toward IBEX. The subsolar region provides only a few percent of J_ENAcomputed_ because most of the cylindrical element is in the magnetosphere and therefore does not contribute to the observed ENA flux.

**Table S1:** Quantities for the 21 cylindrical elements used in the sum in the right hand side of Equation S1.

| Pre-compression (0230-0330 UT): Δl = 1 R_E_, R_0_ = 10 R_E_, ion flux at 0.71 keV (= 369 km/s) = 3.63x10^7^ 1/(cm^2^ s sr keV), σ(0.71 keV) = 1.7x10^-15^ cm^2^, ENA flux (0.71 keV) = 5.65x10^3^ 1/(cm^2^ s sr keV) | | | | | |
| --- | --- | --- | --- | --- | --- |
| Distance from the noon meridian along the IBEX LOS | Percent of cylindrical element in the magnetosheath or boundary layer | Average radial distance from Earth (R_E_) | Model ratio of density/density at the subsolar point | Bulk Velocity along the IBEX LOS (km/s) | Right hand side of (2) = computed ENA flux * n_H0_(10 R_E_) (1/cm^2^ s sr keV)(cm^-3^) |
| 9.5 to 10.5 | 100 | 12.01 | 0.762 | 198.3 | 1.23E+02 |
| 8.5 to 9.5 | 100 | 11.19 | 0.748 | 204.9 | 1.00E+02 |
| 7.5 to 8.5 | 83 | 10.63 | 0.776 | 204.2 | 7.37E+01 |
| 6.5 to 7.5 | 67 | 10.15 | 0.787 | 203.0 | 5.22E+01 |
| 5.5 to 6.5 | 49 | 9.81 | 0.877 | 173.6 | 3.46E+01 |
| 4.5 to 5.5 | 45 | 9.33 | 0.900 | 159.4 | 2.66E+01 |
| 3.5 to 4.5 | 38 | 9.01 | 0.928 | 139.0 | 1.93E+01 |
| 2.5 to 3.5 | 31 | 8.80 | 0.954 | 103.5 | 1.30E+01 |
| 1.5 to 2.5 | 24 | 8.72 | 0.976 | 73.2 | 8.78E+00 |
| 0.5 to 1.5 | 17 | 8.75 | 0.988 | 39.2 | 5.43E+00 |
| -0.5 to 0.5 | 10 | 8.95 | 0.995 | 15.2 | 3.04E+00 |
| -0.5 to -1.5 | 19 | 8.82 | 0.986 | -45.0 | 3.90E+00 |
| -1.5 to -2.5 | 28 | 8.81 | 0.973 | -79.2 | 4.52E+00 |
| -2.5 to -3.5 | 33 | 9.01 | 0.952 | -111.0 | 4.42E+00 |
| -3.5 to -4.5 | 37 | 9.34 | 0.923 | -136.0 | 4.36E+00 |
| -4.5 to -5.5 | 41 | 9.74 | 0.902 | -151.8 | 4.66E+00 |
| -5.5 to -6.5 | 49 | 10.15 | 0.889 | -158.1 | 5.85E+00 |
| -6.5 to -7.5 | 55 | 10.67 | 0.789 | -199.6 | 4.36E+00 |
| -7.5 to -8.5 | 80 | 10.87 | 0.779 | -201.2 | 6.50E+00 |
| -8.5 to -9.5 | 80 | 11.64 | 0.618 | -151.4 | 1.06E+01 |
| -9.5 to -10.5 | 80 | 12.44 | 0.648 | -145.7 | 1.44E+01 |

Table S3 IBEX-Hi and -Lo ENA fluxes for the two time periods before and after the solar wind compression. The error bars represent counting statistics for IBEX-Lo and a 20% error on the absolute fluxes for IBEX-Hi.

| Energy Step | Center Energy (keV) | 0230-0330 UT Flux 1/(cm^2^ s sr keV) | 0400-0515 UT Flux  1/(cm^2^ s sr keV) |
| --- | --- | --- | --- |
| IBEX-Hi 2 | 0.71 | 5.6x10^3^ ±1.1x10^3^ | 1.8x10^4^ ±3.6x10^3^ |
| IBEX-Hi 3 | 1.11 | 2.7x10^3^ ±5.3x10^2^ | 1.3x10^4^ ±2.6x10^3^ |
| IBEX-Hi 4 | 1.74 | 1.5x10^3^ ±3.0x10^2^ | 6.0 x10^3^ ±1.2x10^3^ |
| IBEX-Hi 5 | 2.73 | 7.2x10^2^ ±1.4x10^2^ | 2.9 x10^3^ ±5.9x10^2^ |
| IBEX-Hi 6 | 4.29 | 3.3x10^2^ ±6.5x10^1^ | 1.2 x10^3^ ±2.4x10^2^ |
| IBEX-Lo 1 | 0.015 | 1.2x10^6^ ±1.1x10^6^ | - |
| IBEX-Lo 2 | 0.029 | 2.2x10^5^ ±2.2x10^5^ | 3.2x10^5^ ±2.4x10^5^ |
| IBEX-Lo 3 | 0.055 | 1.5x10^5^ ±1.1x10^5^ | 2.0x10^5^ ±1.2x10^5^ |
| IBEX-Lo 4 | 0.11 | - | 1.5x10^5^ ±6.8x10^4^ |
| IBEX-Lo 5 | 0.209 | 9.2x10^3^ ±1.4x10^4^ | 3.1x10^4^ ±2.3x10^4^ |
| IBEX-Lo 6 | 0.439 | - | 2.3x10^4^ ±1.2x10^4^ |
| IBEX-Lo 7 | 0.872 | 3.7x10^3^ ±2.6x10^3^ | 8.3x10^3^ ±3.5x10^3^ |
| IBEX-Lo 8 | 1.821 | 1.7x10^3^ ±9.8x10^2^ | 1.1x10^4^ ±2.2x10^3^ |

**References:**

Fuselier, S. A., H. O. Funsten, D. Heirtzler, P. Janzen, H. Kucharek, D. J. McComas, E. Möbius, T. E. Moore, S. M. Petrinec, D. B. Reisenfeld, N. A. Schwadron, K. J. Trattner, and P. Wurz (2010), Energetic neutral atoms from the Earth’s subsolar magnetopause, *Geophys. Res. Lett., 37,* L13101, doi:10.1029/2010GL044140, 2010.

Fuselier, S. A., F. Allegrini, M. Bzowski, M. A. Dayeh, M. Desai, H. O. Funsten, A. Galli, D. Heirtzler, P. Janzen, M. A. Kubiak, H. Kucharek, W. S. Lewis, G. Livadiotis, D. J. McComas, E. Möbius, S. M. Petrinec, M. Quinn, N. Schwadron, J. M. Sokół, K. J. Trattner, B. E. Wood, P. Wurz (2014), Low energy neutral atoms from the heliosheath, *Astrophys. J., 784:89*, doi:10.1088/0004-637X/784/2/89.

Petrinec, S.M., Russell, C.T. (1997), Hydrodynamic and MHD equations across the bow shock and along the surface of planetary obstacles, *Space Science Reviews* **79,**757–791, https://doi.org/10.1023/A:1004938724300.

**Figures:**


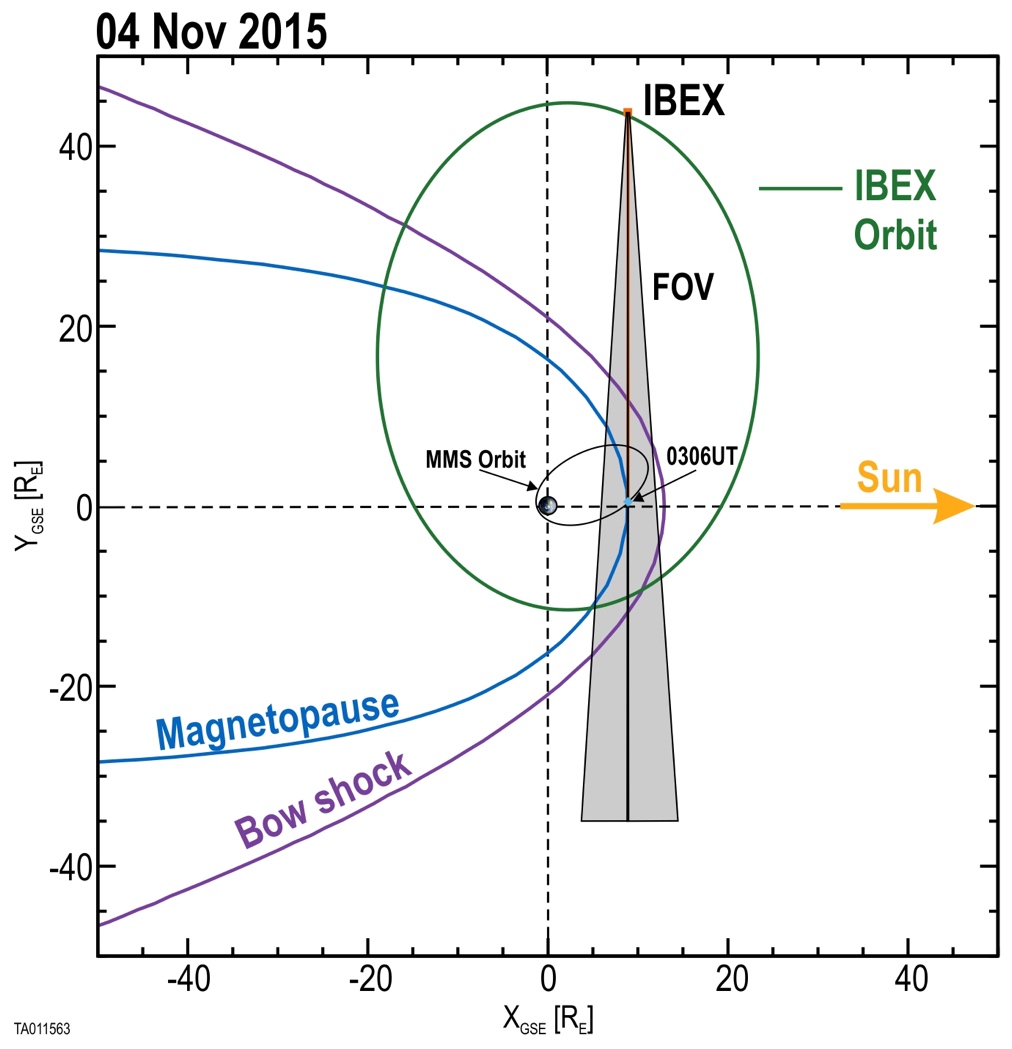


**Figure S1:** Spacecraft configuration on 4 November 2015. IBEX was on the dusk flank with imaging of the subsolar magnetopause. MMS was on its outbound orbit and crossed the magnetopause at a radial distance of 9.3 RE almost exactly at the subsolar point.

**Figure S2:** Six hours of omni data (solar wind data convected to the bow shock) on 4 November 2015. Top to bottom are the total magnetic field, the solar wind density, and the solar wind dynamic pressure. The solar wind dynamic pressure was somewhat elevated but relatively constant during the 3 hours prior to the solar wind compression. The compression occurred at 0400 UT at the bow shock. The total magnetic field increased by almost a factor of 2. However, the change in the other solar wind parameters was much more dramatic. The solar wind density increased by more than a factor of 3, as did the solar wind dynamic pressure. Two time periods were selected to represent conditions before and after the compression.

Figure S3: Schematic of the cylindrical elements along the IBEX LOS as viewed from the Sun and from the north. These elements are used in the sum in (2) for the pre-compression interval. Since the LOS is nearly along constant X_GSE_, the IBEX location is adjusted so that to X’ = 6.7 R_E_ and all cylindrical elements are along X’. Each cylindrical element is described by 5 points in the center, and X and Z’ directions. These points are used to determine the percentage of the cylindrical element that is in the magnetosheath or boundary layer.
